# Can Prevention-Oriented Communication Via Health Organization Websites Affect Adherence to Breast and Cervical Cancer Screening? An Exploratory Study in Italy

**DOI:** 10.34172/ijhpm.9140

**Published:** 2026-03-10

**Authors:** Francesca Conte, Daniela Siano, Rosa Oro

**Affiliations:** ^1^Department of Political and Communication Sciences, University of Salerno, Fisciano, Italy.; ^2^Department of Medicine, Surgery and Dentistry, University of Salerno, Fisciano, Italy.

**Keywords:** Screening Adherence, Web-Based Communication, Health Organizations’ websites, Female Cancers, Italy

## Abstract

**Background:**

Public organizations in healthcare are progressively adopting digital tools to disseminate information regarding the benefits of screening. Nevertheless, no research has hitherto been conducted to investigate the link between the website-based health communications and cancer screening uptake. This study addresses this gap by assessing whether health communications conveyed via the websites of local health organizations improve adherence to breast and cervical cancer screening.

**Methods:**

We performed thematic content analysis of 97 websites belonging to Italian health organizations based on the OSEC-p model. This latter was developed to measure the compliance of prevention-oriented health communication with the effectiveness requirements indicated in the model. Next, correlation and regression analyses were performed to examine the relationship between the average regional OSEC-p scores and the regional breast and cervical cancer screening adherence rates reported by the National Centre for Screening Monitoring in 2022.

**Results:**

The findings show that the prevention-oriented health communication via health organizations’ websites can explain the 45% of the variance in female cancer (breast and cervical) screening uptake. Patient engagement tools and information on preventive cancer initiatives are key web-based communication elements that enhance cancer screening adherence.

**Conclusion:**

This exploratory study is one of the first to highlight the importance of digital health communications for improving cancer screening and public health. It offers insights both for national health policy-makers to optimize health economics through screening campaigns and health organizations managers to enhance the compliance of health communication on websites with the effectiveness requirements defined in the OSEC-p model.

## Introduction

**Key Messages**

**Implications for policy makers**

- This research helps to increase the awareness of health policy-makers about the importance of digital communication for the success of secondary prevention.
- Health policy-makers should strengthen prevention efforts by using websites to increase the population’s health literacy.
- Using validated tools is crucial to accurately measure and evaluate the health communication conveyed through websites.
- Benchmarking can stimulate useful emulation mechanisms for managers of local health organizations that most need to bring about iimprovements in adherence to cancer screening.
- To increase the compliance of prevention-oriented health communication with the effectiveness requirements indicated in the OSEC-p model, it is important to provide detailed information about prevention initiatives on websites through visible, clear, complete, consistent, and accurate communications.

**Implications for the public**

By providing clear, accessible information online about cancer screening, health websites can reach a wider audience and support informed decision-making. Empowering women with accurate knowledge allows them to make thoughtful decisions about their health, such as whether to undergo cancer screening. Educated individuals with a better understanding of the risks and benefits are more inclined to adopt preventive behaviours, including regular screening. This proactive approach not only encourages healthier choices, but also leads to early detection, which improves outcomes and reduces cancer-related mortality. Websites can be an effective tool to bridge information gaps, providing tailored content and interactive resources to improve patient engagement.

Health information dissemination represents a key strategy for fostering preventive healthcare actions within public agenda.^1^ Studies have shown that effective health-related communication improves patient satisfaction, adherence to medical guidelines, enhancing health literacy and public health.^2^ It also increases public awareness of cancer prevention and diagnosis approaches.^3^ Specifically, health information seeking is widely practiced for cancer screening in gynaecological and obstetric field.^4,5^

 Breast and cervical cancers are the cancer with the highest prevalence among women globally, with diagnosis rates of 24.5% and 6.5% and mortality rates of 15.5% and 7.7%, respectively.^6^ In Europe, breast cancer is the most frequently identified cancer, with approximately 380 000 new cases reported in 2022, accounting for 13.8% of all new cancer cases.^7^ Among women aged 15 to 44 years, cervical cancer ranks as the third most prevalent cancer, accounting for around 58 169 new cases diagnosed annually in Europe.^8^ If detected early, the probability of cancer death and the related negative costs can be considerably diminished.^9^

 Given the high prevalence of these cancers, screening services are promoted as essential actions of public health programs. Screening is, in fact, a public health policy, that is a process to control the disease with actions involving the population and clinical components.^10^ In accordance with evidence-based screening plans and European guidelines,^11^ most European Union (EU) countries implement cancer screening as a national public health policy. In Italy, screening programs for female cancers have been promoted since the 1990s and are provided by local health organizations at the regional level.^12^ These programs involve free screenings of eligible women and systems to monitor outcomes.^13^

 Studies have shown that providing accurate information can improve people’s attitudes toward preventive cancer screening.^14,15^ The widespread use of the internet has provided users with greater availability of health information, supporting informed decision-making.^16^

 Many internet users search for health information online to access it more quickly and comprehensively, which can influence their health behaviors and interactions with healthcare providers. In Italy, younger women and people with chronic illnesses are the most active in using the internet for health-related purposes.^17^ Search for health information online has increased significantly in the context of COVID-19.^17^ According to D’Andrea et al^19^ study, online health information–seeking behavior is significantly affected by gender, educational level, and health status.

 Given the opportunities provided by information and communication technology in healthcare sector, public institutions are increasingly adopting digital tools to communicate the advantages of screening.^20^ Indeed, women’s adherence is critically contingent upon their awareness of the program’s existence, so screening uptake increases in response to a greater flow of information.^21^ Among online technologies, websites are particularly useful for seeking health-related information, and for promoting cancer screening.^22,23^

 Despite the increasing attention to these issues, no studies to date have investigated the link between the website-based health communications and cancer screening adherence in the field of gynaecology and obstetrics. We aim to fill this gap by investigating whether and how health communications conveyed via the websites of local health organizations affect adherence to breast and cervical cancer screening.

 In order to achieve this goal, we first conducted thematic content analysis of the websites of 97 local Italian health organizations to evaluate the compliance of prevention-oriented health communication with the effectiveness requirements indicated in the OSEC-p model. Then, using correlation and linear regression analysis, we investigated the relationship between the average regional OSEC-p scores and the regional breast and cervical cancer screening adherence rates reported by the National Centre for Screening Monitoring (Osservatorio Nazionale Screening, ONS) in 2022.

 This exploratory study contributes to the ongoing debate about the potential of digital health communications to improve public health. It also offers insights for both policy-makers of national health system (macro level) and managers of local health organizations (micro level).

## Background

 Health communication focuses on the process of sharing information to motivate public audiences regarding health-related issues.^24^ Communication programmes and public education campaigns are key tools in the health policy development process.^25^ Nutbeam^26^ highlights the importance of health communication in encouraging adherence to medical recommendations and cancer screening.

 Protection motivation theory^27^ suggests health communication can convey cancer risks and highlight the benefits of engaging in preventive behavior. Particularly, the tendency to seek health information online has been linked to adherence to cancer screening.^4^ Indeed, the Internet has transformed the way health information is shared, including cancer screening communication.^28^

 Health communication in digital environments is essential to strengthen motivations for participation in screening programs. Among online technologies, websites are particularly useful for seeking health-related information, and for promoting cancer screening.^23^ Several studies have focused on evaluating health communication on websites in the gynecology and obstetrics fields. Selman et al^22^ examine websites dedicated to sharing information about cervical cancer prevention, assessing credibility of source and accuracy of treatment information. Whitten et al^29^ evaluate websites providing information on breast cancer, considering some elements such as information validity, and literacy content. To assess the health communication of websites intended to encourage screening for breast and cervical cancer, Conte et al^30^ propose the OSEC-p model. It investigates four dimensions: orientation (organization’s commitment to prevention), structure (tools for stakeholder engagement), ergonomics (website quality), and content (cancer prevention actions). Therefore, several studies highlight that digital health communication conveyed through websites must comply with quality and effectiveness criteria to improve cancer screening and achieve health equity.^2^

 Although there is growing attention on these issues, no studies have yet explored whether the prevention-oriented health communication has a relationship with the adherence to cancer screening. Earlier studies have principally concentrated on the function of mass media campaigns to inform the public about the benefits of medical checks.^31^ Some empirical evidence show that marketing campaigns can improve public awareness of cervical screening and mammograms.^32,33^ The impact of online activities on preventive health behaviours has been explored in isolated studies.^34,35^ Nevertheless, these studies have focused on digital technology rather than the websites of local health organizations. Therefore, we pose the subsequent research questions:

 RQ1: Is there a link between prevention-oriented communication via the websites of health organizations and adherence to screening for breast and cervical cancer?

 RQ2: Which dimensions of health organization website communications affect adherence to screening for breast and cervical cancer?

## Methods and Instruments

 Our research design is divided into two phases. In the first phase, we conducted thematic content analysis of the websites of local Italian health organizations to examine health communications for the secondary prevention of gynaecological and obstetric cancers. Thematic content analysis involves the identification, analysis and report of themes that emerge from data.^36^ In this study, thematic content analysis assesses the compliance of prevention-oriented health communication on websites with the effectiveness requirements of the OSEC-p model.^30^ The latter is a tool for evaluating health communications designed to improve adherence to breast and cervical cancer screening. The OSEC-p model was named for its dimensions (macro-items: orientation, structure, ergonomics, and content) and its reference to “prevention” in healthcare. Effectiveness requirements for the prevention-oriented health communication concern the presence on website of a set of micro-items within the four macro-dimensions. Specifically, orientation refers to the organization’s strategic commitment to preventive health, expressed in its mission statement, vision and ethical principles. Structure refers to the credibility of information, tools to encourage stakeholder engagement in screening and the existence of units and functions responsible for cancer prevention. Ergonomics relates to the accessibility, navigability, usability and interactivity of the website and the use of multimedia to facilitate consultation of information on cancer screening. Content refers to information about breast and cervical cancer prevention initiatives and conformity with the communication principles of visibility, authenticity, accuracy, clarity, consistency, and completeness.

 The final OSEC-p score (on a scale of 0–100) is derived from micro-level items, which are treated as dichotomous variables. A score of 1.5625 is the value assigned for the detection of a micro-item, calculated by dividing the maximum obtainable score of 100 by the total number of micro-items, which is 64. Thus, each macro-item spans a range of possible scores, with the upper bound given by the sum of the related micro-item scores: orientation (0–6.2500 based on 4 micro-items), structure (0–18.7500 based on 12 micro-items), ergonomics (0–29.6875 based on 19 micro-items) and content (0–45.3125 based on 29 micro-items).^30^

 Our exploratory study analysed the universe of Italian public health organizations at a local level, except for organizations located in Lombardy and Valle d’Aosta, since the regional rates of adherence to breast and cervical cancer screening for both regions were not in the 2022 ONS report. Thus, our study examined the websites of 97 local health organizations across 19 Italian regions (See Supplementary file 1). The local health organizations in each region were identified by consulting data the Ministry of Health website.^37^ Approximately 6208 micro-items were detected by assessing the presence of 64 specific elements across the websites of 97 local health organizations.

 We focused on the Italian context for several reasons. First, the rate of breast cancer among Italian women is high, in line with other European countries, but with some epidemiological specificities that include the high survival rate. In fact, according to the Italian Association of Medical Oncology,^38^ with a five-year survival rate of 87%-88%, Italy performs better than the European average for breast cancer (82%). This is also thanks to early diagnosis made possible by screening programs. Breast cancer accounted for more than 55 000 new diagnoses in 2022, 30% of female cancers.^39^ In Italy, around 3152 cases of cervical cancer are made each year, making it the fourth leading cancer among young women.^8^ Rates of recovery following a diagnosis of breast or cervical cancer are 86% and 64%, respectively.^39^

 Thus, compliance with cancer screening programmes is vital for lowering cancer-related fatalities in Italy. Since the national public health system was decentralised, the responsibility for cancer screening has been transferred to the Italian regions. While regional inequalities in female cancer screening persist,^40^ participation rates are close to those of the EU.^41^ Moreover, the overarching national system is progressively being improved in line with the provisions of the National Recovery and Resilience Plan and according to “health in all policies” principles. Specifically, accordance with Europe’s Beating Cancer Plan, there is a particular focus on efforts to prevent cancer, starting with strengthening cancer screening programmes.^42^

 Thus, following the OSEC-p criteria, we examined 97 websites belonging to local Italian health organisations, categorised by region. Within each region, we calculated the regional average OSEC-P score, which indicates the degree to which health communication for the prevention of breast and cervical cancer complies with the effectiveness requirements set out in the model. This overall score represents the sum of the mean scores derived from the model’s macro-items: orientation, structure, ergonomics, and content. Content analysis of the websites of Italian health organizations was conducted from May to December 2021. Specific coding was developed to help researchers identify website items and limit subjective interpretations.^30^ This coding is for trained researchers (coders) in accordance with Krippendorff, while the results are intended for managers of local health organizations. Independent parallel analyses were conducted by a public management researcher with minimal prior knowledge of the subject and a medical expert (director of an undergraduate program in obstetrics). Intercoder reliability was 0.82, which is considered satisfactory.^43^

 The second phase was aimed at verifying whether health communications conveyed via the websites of local health organizations influenced cancer screening adherence in Italy. To this end, we first used Pearson’s correlation coefficient to test the relationship between the average regional OSEC-p scores obtained through the thematic content analysis of 97 websites and the average regional screening adherence rates for breast and cervical cancers derived from the 2022 report of the ONS. The average regional rates of ONS report derive from the mean of percentages of adherence to breast and cervical cancer screenings (See Table 1).

**Table 1:** Average Regional Uptake of Breast and Cervical Cancer Screening in Italy

**Regions**	**ONS Report 2022**		
	**Breast Cancer Screening (%)**	**Cervical Cancer Screening (%)**	**Average Between Cancer Screenings (%)**
Emilia Romagna	75	67	71.00
P.A. Trento	73	67	70.00
Friuli Venezia Giulia	70	67	68.50
Piedmont	53	56	54.50
Veneto	64	56	60.00
P.A. Bolzano	48	61	54.50
Liguria	65	42	53.50
Toscany	75	63	69.00
Umbria	74	70	72.00
Marche	46	50	48.00
Lazio	51	38	44.50
Basilicata	55	47	51.00
Sardinia	41	48	44.50
Sicily	52	47	49.50
Abruzzo	36	32	34.00
Molise	35	21	28.00
Calabria	13	30	21.50
Apulia	41	48	44.50
Campania	19	16	17.50

Abbreviation: ONS, Osservatorio Nazionale Screening. Note: The Italian regions considered are 19 since the regional screening adherence rates for breast and cervical cancers for Lombardy and Aosta Valley are not available in the 2022 ONS report. Source: Our elaboration on ONS report.

 Adherence to organized screening initiatives has been measured through a proxy indicating whether or not a woman has paid for screening.^44^ Payment refers to both the full cost of the examination and the ticket only. Therefore, “women examined” refers to those women who participated after receiving an invitation and for whom the diagnostic/therapeutic process was recovered. In this study, adherence outside of organized screening (opportunistic screening) was not considered because this study was aimed at measuring the influence of local health organization communications on screening adherence. Then, this study conducted a linear regression analysis to explore how the prevention-oriented communication, assessed through OSEC-p model, and its dimensions (orientation, structure, ergonomics, content), affected cancer screening adherence. The software used to run the correlation analysis was IBM SPSS (Statistical Package for Social Sciences) version 23.

 As already pointed out, the data used in the present study were obtained from the Italian National Screening Observatory which provides exclusively aggregated regional-level adherence rates expressed as percentages. Individual-level binary data on participation are not publicly available; therefore, it was not possible to model screening adherence using individual-level binary outcomes.

 Although adherence was expressed as proportions, the regional ONS percentages showed a distribution sufficiently close to normality, as assessed empirically, to justify the use of linear regression without substantial violations of model assumptions.

 For these reasons, the use of linear regression for the analysis of aggregated public health data was considered an appropriate approach, given that it is common practice in epidemiological studies, especially when proportions are calculated on very large populations, as is the case in national screening programs.

## Results

 The results of Italian inter-regional benchmarking show that the websites of healthcare organizations in the Umbria region are more compliant with the effectiveness requirements indicated in the OSEC-p model (See Table 2).

**Table 2:** Italian Inter-regional Benchmarking Based on Average Regional OSEC-p Scores

**Italian Regions**	**Average Orientation (0-6.2500)**	**Average Structure ** **(0-18.7500)**	**Average Ergonomics** **(0-29.6875)**	**Average Content ** **(0-45.3125)**	**Average Score OSEC-p (0-100)**
Umbria	4.69	14.06	17.97	35.16	71.88
Emilia Romagna	3.71	11.33	19.73	28.32	63.09
Veneto	3.47	12.50	18.23	28.47	62.67
Liguria	3.40	10.60	18.10	30.00	62.10
Friuli Venezia Giulia	4.68	10.93	19.79	26.56	61.96
P.A. Trento	3.12	14.06	14.06	29.69	60.93
Toscany	4.20	12.00	17.70	25.90	59.80
Piedmont	4.17	13.28	16.93	25.39	59.77
Lazio	4.06	13.44	14.69	27.19	59.38
Basilicata	3.12	12.50	21.88	20.31	57.81
Campania	4.46	10.94	14.96	27.23	57.59
Sicily	4.69	10.07	15.28	27.08	57.12
P.A. Bolzano	3.12	6.25	18.75	28.12	56.25
Apulia	3.91	8.85	17.71	24.74	55.21
Molise	4.69	7.81	18.75	23.44	54.69
Abruzzo	2.70	7.00	16.40	26.70	52.80
Marche	3.12	10.94	15.62	21.87	51.55
Calabria	4.69	8.44	16.87	18.75	48.75
Sardinia	2.34	7.03	12.89	23.63	45.89

Source: our elaboration.

 Using Pearson’s correlation coefficient, we found a positive association between the prevention-oriented health communication and the cancer screening adherence in gynaecological and obstetric field, addressing what stated RQ1. Indeed, the results show a positive correlation between average regional OSEC-p scores and adherence to breast and cervical cancer screening (*r* = 0.671, *P* < .01). According to Cohen’s effect size guidelines,^45^ *r* ≥ 0.50 is considered strong.

 Then, we performed a bivariate linear regression to test the influence of health communication for secondary prevention (independent variable), measured through the OSEC-p model, on the participation in screening initiatives (dependent variable).

Table 3 indicates that the R^2^ is 0.450 and the regression model is significant (*P* < .01).

**Table 3:** Bivariate Linear Regression Between Regional Means of OSEC-p and Regional Means of Adherence to Breast and Cervical Cancer Screening Reported in the 2022 ONS Report

**Model Summary**						
**Model**	**R**	**R Square**		**Adjusted R Square**	**Standard Error of the Estimate**	
1	0.671	0.450		0.418	12.56524	
**Coefficients**						
**Model**		**Unstandardized Coefficients**		**Standardized Coefficients**	**t**	**Sig.**
		**B**	**Standard Error**	**Beta**		
**1**	(Constant)	-59.280	29.504		-2.009	.061
	OSEC-p	1.894	0.508	0.671	3.732	.002

Abbreviation: ONS, Osservatorio Nazionale Screening. Source: Our elaboration.

 This means that the prevention-oriented health communication via the websites of health organisations explains 45% of the variance in adhering to breast and cervical cancer screening. Thus, the better health communication on websites adheres to the effectiveness requirements defined by the OSEC-p model, the greater the adherence to cancer screening. The regression line is illustrated in Figure.

**Figure:**
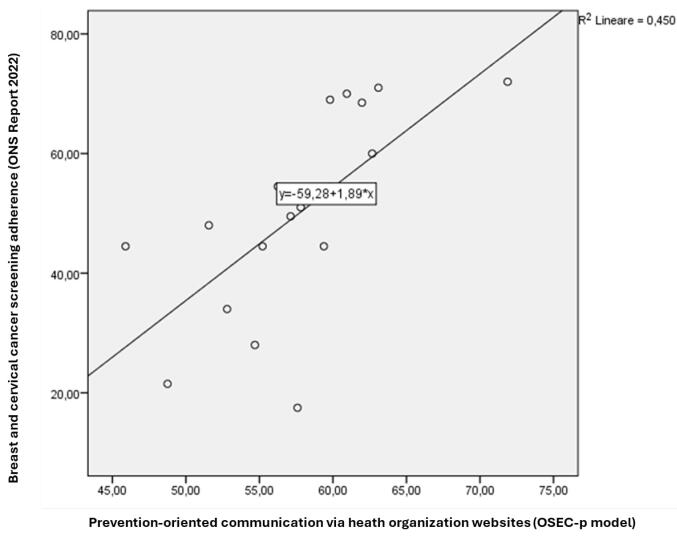
Scatter Plot and Regression Lines. Abbreviation: ONS, Osservatorio Nazionale Screening. Source: Our elaboration.

 RQ2 refers to the dimensions of prevention-oriented health communication via websites. We carried out a multiple linear regression analysis, using macro-items of OSEC-p model (orientation, structure, ergonomics, content) as predictors and adherence to screening programs as dependent variable. The data in Table 4 indicates that the R^2^ is 0.592, and the regression model is regarded as being statistically significant (*P* < .05). The macro-item “structure” and “content” are significant predictor (*P* < .05) of preventive health behaviours.

**Table 4:** Multiple Linear Regression Between Regional Means of OSEC-p Macro-items and Regional Means of Breast and Cervical Cancer Screening Adherence Reported in the 2022 ONS Report

**Coefficients**						
Model		**Unstandardized Coefficients**		**Standardized Coefficients**	**t**	**Sig.**
		**B**	**Standard Error**	**Beta**		
1	(Constant)	-47.773	31.431		-1.520	.151
	Orientation	-6.741	3.950	-0.306	-1.707	.110
	Structure	2.884	1.268	0.431	2.274	.039
	Ergonomics	2.512	1.287	0.340	1.952	.071
	Content	1.903	0.818	0.430	2.326	.036

Abbreviation: ONS, Osservatorio Nazionale Screening. Source: Our elaboration.

 The results highlight that patient engagement and organizational tools dedicated to prevention, as well as the communication of breast and cervical cancer initiatives, can positively affect participation in cancer screening. Otherwise, the strategic orientation toward preventive health is not significant predictor of breast and cervical cancer screening adherence. Ergonomics of website has a positive effect, albeit at the limits of statistical significance.

## Discussion

 This study enriches the research on the factors influencing healthcare behaviours, specifically participation in cancer screening.^40^ The empirical evidence reveals a positive correlation between prevention-oriented communication via the websites of health organizations and breast and cervical cancer screening adherence (RQ1). Approximately 45% of female cancer screening uptake can be explained by the compliance of health organization websites with the effectiveness requirements indicated in the OSEC-p model. This percentage is considered relevant, given that additional factors, such as level of education, socioeconomic status, health literacy, cultural background, geographical disparities, and access to healthcare services,^46^ may affect screening behaviour for the remaining 55%.

 This result is in line with protection motivation theory,^27^ which states health communication plays an important role in reinforcing the motivation behind participation in screening programs, as it sheds light on the risks of not taking preventive action. Findings are consistent with studies highlighting that e-health communications have great potential to promote preventive behaviours.^47,48^ The AIRC Foundation for Cancer Research in Italy emphasises the central role of “mobile health,” the use of smartphones to communicate health-related initiatives via websites Mobile health increases the likelihood of patients adhering to oncological screenings by 50%.^49^ Moreover, Cvijović et al^31^ emphasize the role of the internet in promoting female cancer prevention, showing that 42% of women, especially those of younger generations, seek information about secondary prevention on health websites and one fifth of them on the official sites of medical institutions.

 Further, some studies have examined how online communication can raise awareness related to the risk of developing cancer and emphasize the importance on prevention.^50,51^

 Regarding RQ2, the study highlights that the tools and procedures (eg, web sections dedicated to women, social networks, satisfaction surveys) that improve patient engagement affect screening adherence. These tools promote not only access to information but also a sense of empowerment and involvement in the decision-making process by patients. The literature supports the value of co-design and participatory approaches in health communication. For instance, Pollock et al^52^ demonstrate that actively involving stakeholders improves the quality, value and impact of health-related initiatives. Patient engagement in healthcare is vital for improving information management and diagnostic procedures.^53^

 Moreover, also content on secondary prevention encourage compliance with cancer screening, confirming the importance of disseminating quality and relevant information on gynaecological and obstetric screening initiatives through websites.^29^ It has been shown that screening attendance can be significantly raised by stakeholder engagement, clear communication and the effective use of online tools.^54^

###  Practice Implications

 In terms of management and policy implications, this paper provides insights into the healthcare sector at both the macro and micro levels. From the macro perspective, the results offer evidence to assist health policy-makers in optimizing health economics and management. Indeed, the main purpose of screening studies is to guide health policy decisions.^55^ This research helps to increase the awareness of health policy-makers about the potential of digital communication to facilitate the success of prevention programmes. Health policy-makers should improve health prevention actions through websites to enhance health literacy. By communicating information about cancer screening through websites, health policy-makers should promote informed decision-making that allows women to make conscientious decisions.^56^ In fact, educated individuals are more likely to respond strongly by taking preventive measures, such as getting cancer screening, when they have more information.^57,58^ Our study highlights the importance of measuring the compliance of health communication with the effectiveness requirements of the OSEC-P model, as this can encourage people to adhere to cancer screening programs. Therefore, policy-makers should invest more in well-designed and managed webpages for prevention-oriented health communication.

 Digital health communication, which improve adherence to breast and cancer screening, also has positive effects on the health economy in terms of reduced healthcare costs. Although there are direct costs associated with implementing cancer screening, such as those for information campaigns, logistics, training of health professionals and purchase of equipment,^59^ these costs can be offset if screening results in significant long-term reductions in healthcare costs.^60^ Organised screening is more effective and cost-saving than opportunistic screening.^61^ Indeed, improved the uptake of organized cancer screening, facilitated by proficient health communication on websites, contributes to timely diagnosis of the cancer, which can decrease treatment costs of advanced diseases, such as complex surgery or prolonged hospitalisation. Early diagnosis therefore allows diseases to be treated at an earlier stage, when treatment is less complex and costly, helping to reduce long-term healthcare costs.

 Moreover, health system actors should not only develop effective preventive communication campaigns but also provide protocols and guidelines to improve the effectiveness of local cancer screening campaigns. To support managers of local health organizations, health policy-makers should carry out inter-regional benchmarking to provide useful indications on best digital health communication practices.

 From the micro perspective, the results of the study may encourage managers of local health organizations to pay particular attention to website tools that promote dialogue with women through stakeholder engagement process. Efforts to increase patient engagement strategies in healthcare have been embraced by policy-makers as important drivers to improving patient experience.^62^ To increase the compliance of health communication on websites with the effectiveness requirements indicated in the OSEC-p model, it is also important to provide detailed information about secondary prevention initiatives on websites through visible, clear, complete, and accurate communications. Guidelines, protocols and best practices derived from inter-regional benchmarking of website health communications are essential for managers of local health organizations. These information sources may improve the quality of cancer screening campaigns that are autonomously developed and managed by health organisation managers to benefit specific regions.

 Finally, given that the macro and micro levels must be closely interconnected and in constant dialogue, a national representative should be employed to coordinate the activities of local health organizations.

## Conclusions

 This study is one of the first to investigate the influence of the prevention-oriented communication conveyed via the websites of local health organizations on breast and cervical cancer screening adherence. This research advances the knowledge of the factors that influence cancer screening uptake,^46^ by providing empirical evidence on the importance of health communication as a means of enhancing individuals’ motivation to participate in prevention programs.

 This paper has some limitations that can inform future studies. Although this research was conducted in Italy, its results can support health policies in Europe given that cancer screening participation rates in Italy are in line with those of EU countries.^41^ However, to verify the preliminary results of this study, it would be appropriate to extend our investigation to other European countries to obtain international benchmarking and explore potential cultural, organizational, and policy differences that may influence the health communication strategies.

 Another limitation is that the study is cross-sectional, so it is not possible to assess changes over time. A longitudinal design could be useful to examine how communication strategies evolve and how they impact screening adherence in the medium to long term.

 The study is also limited to the analysis of the corporate website, thus future studies could deepen the health communication conveyed by health organizations through other digital tools, such as social media, which offer interesting ways to communicate health messages, emphasizing cancer prevention. Adopting a multi-platform approach could facilitate an in-depth evaluation of the communication strategies adopted by healthcare institutions.

 Moreover, since our study identifies a correlation between the compliance of prevention-oriented health communication via website with effectiveness requirements and screening adherence, future research, including longitudinal or experimental studies, can explore potential causal relationships.

 It would be interesting to investigate how sociodemographic factors, such as age and education, influence the link between health communication and screening participation.

 Finally, the study focuses on the content and strategies used by healthcare providers without incorporating the perspectives of the target audience. Future research could broaden the scope by exploring the perspectives of communication recipients to verify the way in which users are encouraged to adhere to cancer screening programs.

## Disclosure of artificial intelligence (AI) use

 Not applicable.

## Ethical issues

 Ethics approval was not required as this work only involved analysis of publicly

 available material.

## Conflicts of interest

 Authors declare that they have no conflicts of interest.

## Supplementary files


Supplementary file 1. Italian Regional Health Organizations.
