# Supplementary material for: Can Prevention-Oriented Communication Via Health Organization Websites Affect Adherence to Breast and Cervical Cancer Screening? An Exploratory Study in Italy
Source: Int J Health Policy Manag. 2026 Mar 10;15:9140. doi: 10.34172/ijhpm.9140 (PMC13145250; doi:10.34172/ijhpm.9140)
Supplement: Supplementary file 1 — Italian Regional Health Organizations. [file ijhpm-15-9140-s001.pdf]

Article title: Can Prevention-Oriented Communication Via Health Organization Websites Affect Adherence to Breast and Cervical Cancer Screening? An Exploratory Study in Italy

Journal name: International Journal of Health Policy and Management (IJHPM)

Authors' information: Francesca Conte[1], Daniela Siano[2], Rosa Oro[2]

[1]Department of Political and Communication Sciences, University of Salerno, Fisciano, Italy.

[2]Department of Medicine, Surgery and Dentistry, University of Salerno, Fisciano, Italy.

***Correspondence to:** Francesca Conte; Email: fconte@unisa.it

Citation: Conte F, Siano D, Oro R. Can prevention-oriented communication via health organization websites affect adherence to breast and cervical cancer screening? An exploratory study in Italy. Int J Health Policy Manag. 2026;15:9140. doi:10.34172/ijhpm.9140

Supplementary file 1. Italian Regional Health Organizations

Geographic area	Regions	Local health organizations	Number of websites by region
**North**	Veneto	ULSS<br>Dolomiti, Marca Trevigiana, Serenissima, Oriental Veneto, Polesana, Euganea, Pedemontana, Berica, Scaligera	9
	P.A. Trento	APSS<br>Trento	1
	Piedmont	ASL<br>TO3, TO4, TO5, VC, BI, NO, VCO, CN1, CN2, AT, AL, Torino	12
	Liguria	ASL<br>Genovese, Savonese, Imperiese, Chiavarese, Spezzino	5
	Friuli Venezia Giulia	ASU<br>Giuliano Isontina, Central Friuli, Western Friuli	3
	P.A. BolzanoAlto Adige	AS<br>Alto Adige	1
	Emilia Romagna	AUSL<br>Piacenza, Parma, Reggio Emilia, Modena, Bologna, Imola, Ferrara, Romagna	8
**Center**	Lazio	ASL<br>Rome 1, Rome 2, Rome 3, Rome 4, Rome 5, Rome 6,<br>Latina, Viterbo, Rieti, Frosinone	10

	Toscany	AUSL<br>Center-Tuscany, Northwest-Tuscany, Southeast-Toscany	3
	Umbria	AUSL<br>n.1, n.2	2
	Marche	ASUR<br>Marche	1
**South and Islands**	Abruzzo	ASL<br>Avezzano-Sulmona-L'Aquila, Lanciano-Vasto-Chieti, Pescara, Teramo	4
	Calabria	ASP<br>Cosenza, Crotone, Catanzaro, Vibo Valentia, Reggio Calabria	5
	Campania	ASL<br>Naples, 1 Naples 2, Naples 3, Salerno, Caserta, Benevento, Avellino	7
	Sicily	ASP<br>Agrigento, Caltanissetta, Catania, Enna, Messina, Palermo, Ragusa, Syracuse, Trapani	9
	Sardinia	ASST<br>Sassari, Gallura, Nuoro, Ogliastra, Oristano, Medio Campidano, Sulcis, Cagliari	8
	Apulia	ASL<br>BR, TA, BT, BA, FG, LE	6
	Basilicata	ASL<br>Potenza, Matera	2
	Molise	ASRE<br>Molise	1

Note: The names of local health organizations vary according to the region: ATS is "Agenzia di Tutela della Salute" (Health Protection Agency); ULSS is "Unità Locale Socio Sanitaria" (Local Social Health Unit); AUSL is "Azienda - Unità Sanitaria Locale" (Local Health Unit – Authority); APSS is "Azienda Provinciale per i Servizi Sanitari" (Provincial Health Services Authority); ASL is "Azienda Sanitaria Locale" (Local Health Authority); ASU is "Azienda (University Health Authority); AS is "Azienda Sanitaria" (Health Authority); ASUR is "Azienda Sanitaria Unica Regionale" (Single Regional Health Authority); ASP is "Azienda Sanitaria Provinciale" (Provincial Health Authority); ASST is "Aziende Socio Sanitarie Territoriali" (Territorial Social Health Companies); ASRE is "Azienda Sanitaria Regionale" (Regional Health Authority).

Source: Ministry of Health (2023).
